# *MyGuide long COVID*: An online self-management tool for people with long COVID

**DOI:** 10.1016/j.invent.2025.100825

**Published:** 2025-04-04

**Authors:** Hiten Naik, Kyla Pongratz, Michelle Malbeuf, Sonya Kung, Lori Last, Asuka Sugiyama, Esther Khor, Marlee McGuire, Adeera Levin, Karen C. Tran

**Affiliations:** a Post-COVID-19 Interdisciplinary Clinical Care Network, British Columbia, Canada; b Department of Medicine, The University of British Columbia, Vancouver, Canada; c Provincial Health Services Authority, British Columbia, Canada; d Providence Health Care, Vancouver, British Columbia, Canada

**Keywords:** SARS-CoV-2, Long COVID, Post-acute sequalae of COVID-19, Post-COVID-19 condition, Digital health, Self-management, Chronic disease

## Abstract

**Background:**

Long COVID is a relatively new condition for which patients are asked to employ self-management strategies to manage their symptoms. However, it can be challenging for individuals with long COVID to find reliable and actionable self-management resources. The objective of this project was to develop an online tool for individuals with long COVID that is patient-centered, accessible, and customizable to meet individual needs.

**Methods:**

*MyGuide Long COVID* (www.longCOVIDguide.ca) was developed in British Columbia (BC), Canada, by a team that included long COVID clinicians and patient partners. Site visitors answer questions about their symptoms, and *MyGuide* generates a curated set of self-management resources tailored to their needs. Since its launch in August 2023, Google Analytics has been used to monitor website activity.

**Results:**

Within the first year, MyGuide had 52,578 total page views and 8570 new users. The most popular method to access *MyGuide* was by computer (56.3 % of users), and the most represented city was Vancouver, BC (23.5 % of users). The most popular topics were “Post Exertional Malaise” (1339 sessions) and “What is long COVID?” (1257 sessions).

**Conclusions:**

An online tool to support chronic disease self-management can be successfully co-developed with patient partners and engagement tracked using web analytics.

## Highlights

• It can be challenging for people with long COVID to find actionable self-management resources.
• Developed with input from clinicians and patients, *MyGuide Long COVID* curates a set of resources for site visitors.
• Within the first year, *MyGuide Long COVID* had 52,578 page views and 8570 new users.
• *MyGuide Long COVID* is an example of an online tool can be co-created with patient partners and engagement tracked using web analytics.

## 1 Introduction

It is estimated that approximately 400 million people worldwide have experienced long COVID (also referred to as post-COVID-19 condition and post-acute sequelae of COVID-19), which is defined as persistent symptoms lasting three months or more following a SARS-CoV-2 infection (Soriano et al., 2022; Al-Aly et al., 2024; Ely et al., 2024). Long COVID is associated with over 200 symptoms (Davis et al., 2021). Common symptoms include fatigue, post-exertional malaise, cognitive difficulties (also known as “brain fog”), and shortness of breath (Davis et al., 2023). Self-management has become a central tenet in long COVID care, especially since no widely accepted pharmacological treatment exists yet for this new condition (Décary et al., 2021; Busse et al., 2025). The self-management paradigm for long COVID and other chronic diseases emphasizes the importance of tools and interventions that foster self-efficacy in patients by teaching them about their condition and equipping them with the skills to monitor and manage their symptoms (Busse et al., 2025; Grady and Gough, 2014). Examples of self-management strategies for long COVID include pacing and mindfulness (Décary et al., 2021; Jreidini, 2022; Rybkina et al., 2024). However, it can be challenging for people with long COVID to find reliable, actionable self-management resources due to the disabling nature of long COVID symptomatology, the heterogeneity of long COVID clinical presentations, widespread misinformation online, and the lack of provider knowledge (Davis et al., 2021; Jreidini, 2022; Sule et al., 2023; Kingstone et al., 2020).

We observed these challenges while supporting people with long COVID in British Columbia (BC), Canada, and sought to address them by developing an innovative website called *MyGuide Long COVID*. *MyGuide Long COVID* was inspired by a locally developed website called *MyGuide Concussion*, which people with concussions found validating and helpful in their recovery (Kang et al., 2022). Like *MyGuide Concussion*, we aimed to create a website that would curate self-management resources for users after they had answered a series of questions about their symptoms. With *MyGuide Long COVID,* our objective was to develop an online, customizable tool tailored to the unique needs of the long COVID population and one that is accessible to individuals from diverse backgrounds, geographical locations, and abilities.

## 2 Methods

### 2.1 Team

The development of *MyGuide Long COVID* was led by Provincial Digital Solutions (PDS) from the Provincial Health Services Authority (PHSA) in collaboration with the Post-COVID-19 Interdisciplinary Clinical Care Network (PC-ICCN) in BC. PHSA is a province-wide provider of specialized health services, and PC-ICCN is a network under PHSA that was established to provide long COVID care, education, and research throughout the province (Naik et al., 2023) The *MyGuide Long COVID* team comprised project leads from PDS and PC-ICCN, long COVID clinicians (including physicians, nurses, social workers, occupational therapists, and physiotherapists), communications specialists, a medical writer, a graphic designer, and three patient partners. A web development firm was contracted to build the website.

### 2.2 Development process

The *MyGuide Long COVID* team met weekly to discuss the website's structure, flow, and content, as well as to review prototypes. Long COVID topics were selected for inclusion in *MyGuide Long COVID* based on recommendations from patient partners and drafted by one to two clinicians, informed by the best available evidence and clinical experience. As definitive information about long COVID was sparse, some recommendations were adapted from similar conditions, such as myalgic encephalomyelitis/chronic fatigue syndrome. All content was then reviewed by at least two patient partners and the PC-ICCN Director before being revised and finalized by the medical writer. When engaging patient partners, we implemented best practices in co-creation and co-design (BC Patient Safety & Quality Council and Patient Voices Network, 2022).

### 2.3 Promotion and dissemination

*MyGuide Long COVID* was shared with the long COVID patient community through an e-newsletter and individually at their PC-ICCN clinical appointments. To encourage long COVID clinicians to share *MyGuide Long COVID* with their patients, we developed an auto-text that could be used in the electronic health record. *MyGuide Long COVID* was promoted to primary care providers and the general public through presentations at virtual seminars and conferences. Metadata was strategically developed for search engine optimization (SEO).

### 2.4 Web analytics

We monitored interest in *MyGuide Long COVID* using Google Analytics 4 and followed the methodological guidelines for systematic assessments of healthcare websites using web analytics (Fundingsland EL et al., 2022). We tracked page views, new users, and sessions. We monitored the number of sessions for each *MyGuide Long COVID* topic and examined new user acquisition by location, device, and medium (i.e., how users found the website).

## 3 Results

**Fig. 1:**
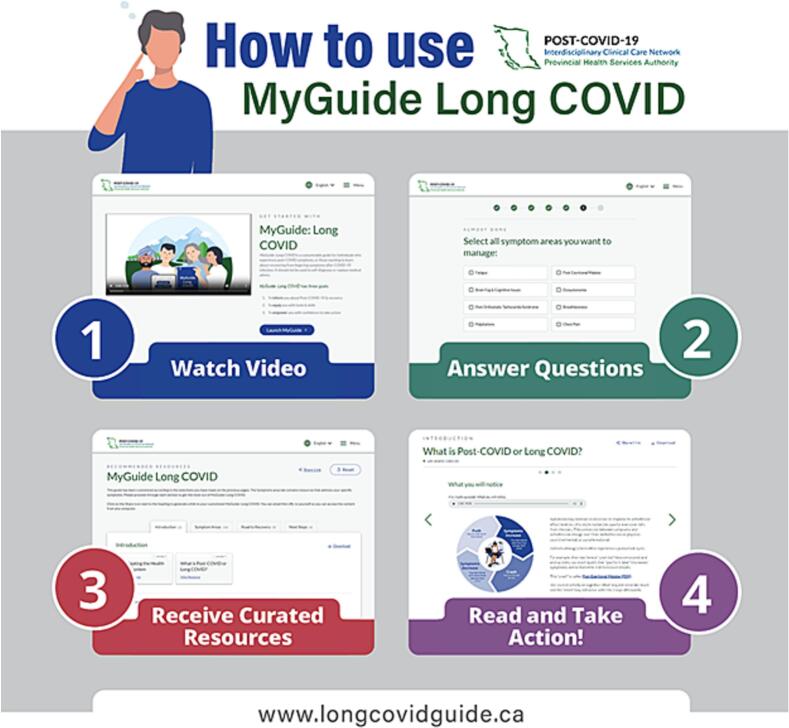
Infographic describing how to use *MyGuide Long COVID*.

When launched on August 10, 2023, *MyGuide Long COVID* comprised 29 topics organized into four categories (**Table S1**). Users accessing the website are presented with a short introductory video and asked seven questions, from which a curated library of topics is generated (Fig. 1). Each topic is presented as a series of frames with minimal text, sometimes accompanied by pictures, diagrams, or video. Users can download and share their customized “guide,” which includes all their topics in Portable Document Format (PDF). Some topics also include downloadable worksheets. *MyGuide Long COVID* is available in dark mode and audio format. *MyGuide Long COVID* was initially available in English, but has since been translated to Punjabi, Tagalog, Simplified Chinese, Traditional Chinese, and French.

**Figure S1:**
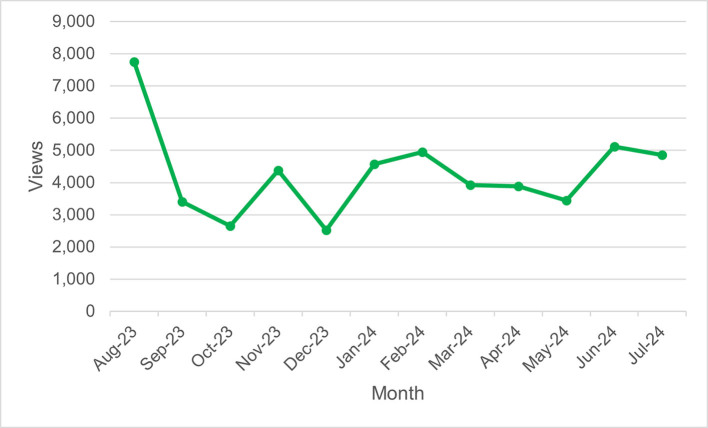
*MyGuide Long COVID* page views over time

**Figure S2:**
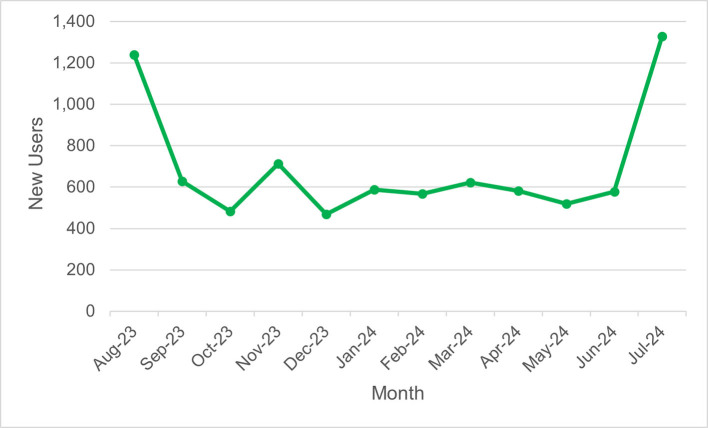
*MyGuide Long COVID* new users over time

*MyGuide Long COVID* had a total of 52,578 page views, 8559 new users, and 16,256 sessions. In general, activity was highest in the first month before declining and relatively steady throughout the year (Figure S1**;** Figure S2). Of the users, 23.5 % were from Vancouver (**Table S2**). The most popular device for accessing MyGuide Long COVID was a computer (56.3 % of users) (**Table S3**), and the most common medium was direct traffic (**Table S4)**. The most popular topics were “Post Exertional Malaise” and “What is long COVID?” (**Table S5**).

## 4 Discussion

People with long COVID have difficult, often unexplained symptoms, but can experience challenges accessing support (Naik et al., 2024; Baz et al., 2023; Karpman et al., 2023; Goodridge et al., 2023; MacPherson et al., 2022; Turk et al., 2024). With *MyGuide Long COVID*, we leveraged the accessibility of online media to address the urgent need for a long COVID support tool. Lessons from our experience can be applied to developing digital self-management tools for other chronic conditions.

### 4.1 Lesson 1: Importance of patient involvement

Patient partners played a crucial role in ensuring that the *MyGuide Long COVID* user experience was relevant and tailored to the needs of individuals with long COVID. For example, incorporating dark mode and audio recordings directly addressed concerns from patient partners regarding photosensitivity and the “information overload” experienced by some individuals with long COVID. Patient partners also ensured that we used validating and actionable language and that topics were displayed in order of relevance. Patient partner co-design has played an essential role in the development of other non-pharmacological interventions for long COVID. For example, the Long Covid Personalised Self-managemenT support EvaluatioN (LISTEN) clinical trial investigated a self-management strategy that was developed in partnership with people living with long COVID (Busse et al., 2025).

### 4.2 Lesson 2: Interdisciplinary collaboration

Published reports have emphasized the value of involving an interdisciplinary team of health professionals in long COVID care, which often involves both pharmacological and non-pharmacological treatment approaches (Jreidini, 2022; Naik et al., 2023; Levin et al., 2022; Dean, 2023; Chou et al., 2024). The development of *MyGuide* mirrored this philosophy. Clinicians representing multiple health professions contributed to the content, resulting in balanced and holistic recommendations for symptom self-management.

### 4.3 Lesson 3: Utilization of web analytics

Web analytics enabled the real-time evaluation of MyGuide uptake, allowing us to gauge the impact of dissemination strategies. For example, we observed an increase in new users in July 2024, following the promotion of the initiative to organizations outside of BC (Figure S2).

### 4.4 Limitations and future directions

Through analysis of web analytics data and informal feedback, we recognized several limitations of *MyGuide* and plan to address these in our next update. First, the original *MyGuide* content was limited by the paucity of published treatment options and guidelines for long COVID; this will be addressed by integrating the forthcoming Canadian Guidelines on Post COVID-19 Condition. Second, we recognized that caregivers of people with long COVID play a crucial role in long COVID care (Cavalcante et al., 2023), and our next update will include content for these users. Third, we will expand from being a BC-focused tool to one with a national scope. To achieve this, we have added a French language option and following a survey of the long COVID community across Canada, we have changed the web address from www.longcovidbc.ca to www.longcovidguide.ca. Lastly, we recognize that additional quantitative and qualitative feedback from users is required to evaluate *MyGuide Long COVID*. As such, we have developed a short feedback survey that is now readily accessible from all pages of the website.

## 5 Conclusions

Chronic disease management can and should offer alternatives to in-person care. Following the format of *MyGuide Concussion*, *MyGuide Long COVID* enhances existing care by offering online self-management resources that can be accessed anytime from any device. We expect that *MyGuide Long COVID* will empower and provide autonomy to individuals with long COVID, thereby reducing the burden on care providers. The sustained volume of new users over the course of a year is an indication that this online tool is addressing a need in the long COVID community. With an emphasis on patient-centredness, accessibility, interdisciplinary collaboration, and continuous evaluation, we anticipate that our approach can also be successfully applied to other chronic conditions.

The following are the supplementary data related to this article.

Supplementary tables Image 1

## Funding/ support

Hiten Naik has been supported by The University of British Columbia Clinician Investigator Program, a CAN-TAP-TALENT & Michael Smith 10.13039/100005622Health Research BC Postdoctoral Fellowship, and a Post-COVID-19 Interdisciplinary Clinical Care Network (PC-ICCN) Research Fellowship. The PC-ICCN is a partnership between the provincial Ministry of Health, Provincial Health Services Authority (PHSA), Providence Health Care, B.C.'s regional health authorities, patients, and research organizations across B.C. It has received research funding from the St. Paul's Foundation. *MyGuide Long COVID* is an initiative funded by PHSA. The inclusion of the Canadian Guidelines for Post COVID-19 Condition has been made possible through a financial contribution from the Public Health Agency of Canada. The views expressed herein do not necessarily represent the views of the Public Health Agency of Canada.

## Role of funder

The funders had no role in the collection, management, analysis, and interpretation of the data, the preparation, review, or approval of the manuscript, or the decision to submit the manuscript for publication.

## Declaration of competing interest

The authors declare the following financial interests/personal relationships which may be considered as potential competing interests: Hiten Naik is a Guideline Team member for the Canadian Guidelines for Post-COVID-19 Condition. The other authors declare that they have no known competing financial interests or personal relationships that could have appeared to influence the work reported in this paper.
