# Supplementary material for: MyGuide long COVID: An online self-management tool for people with long COVID
Source: Internet Interv. 2025 Apr 4;40:100825. doi: 10.1016/j.invent.2025.100825 (PMC12008151; doi:10.1016/j.invent.2025.100825)
Supplement: Supplementary file 1 — Supplementary tables [file mmc3.docx]

***Supplemental Material***

*Naik et al. MyGuide Long COVID*: An online self-management tool for people with long COVID

**Table of Contents**

[**Table S1: Outline of *MyGuide Long COVID* content** 2](#_Toc194607979)

[**Table S3: New users by device** 4](#_Toc194607980)

[**Table S4: New users by medium** 5](#_Toc194607981)

[**Table S5: *MyGuide Long COVID* sessions by topic** 6](#_Toc194607982)

# **Table S1: Outline of *MyGuide Long COVID* content**

| **Categories** | **Topics** |
| --- | --- |
| 1. Introduction | Navigating the Health Care System |
|  | What is Post COVID-19 or long COVID? |
|  | Diagnosis of long COVID |
| 2. Symptom Areas | Brain Fog |
|  | Breathlessness |
|  | Chest Pain |
|  | Dizziness |
|  | Dysautonomia |
|  | Fatigue |
|  | Hair Loss |
|  | Headaches |
|  | Mental Health |
|  | Palpitations |
|  | Post Exertional Malaise |
|  | Postural Orthostatic Tachycardia Syndrome (POTS) Management |
|  | Ringing in the Ears |
|  | Taste and Smell Changes |
| 3. Road to Recovery | Breath Work |
|  | Energy Conservation |
|  | Heart Rate Monitoring |
|  | Nutrition |
|  | Pacing |
|  | Return to Physical Activity |
|  | Return to Work |
|  | Sleep Hygiene |
|  | Tools and Worksheet |
| 4. Next Steps | Becoming a Self Manager |
|  | Becoming an Informed Consumer |
|  | Participate in COVID-19 Research |
|  | What is PC-ICCN? |

**Table S2: Locations of new users of *MyGuide Long COVID***

| **Location** | **New Users (%)** |
| --- | --- |
| Vancouver, British Columbia | 2,013 (23.5) |
| Toronto, Ontario | 519 (6.1) |
| Unknown location | 432 (5.0) |
| Victoria, British Columbia | 260 (3.0) |
| Surrey, British Columbia | 228 (2.7) |
| Kelowna, British Columbia | 210 (2.5) |
| Quebec City, Quebec | 199 (2.3) |
| Burnaby, British Columbia | 163 (1.9) |
| Calgary, Alberta | 137 (1.6) |
| Nanaimo, British Columbia | 124 (1.4) |

**Legend:** This table lists the number of new users of *MyGuide Long COVID* from August 10, 2023, to August 9, 2024. The top 10 locations in terms of new users are listed. The percentages represent the proportion of total new users during this period (8,570).

# **Table S3: New users by device**

| **Device** | **New Users (%)** |
| --- | --- |
| Computer | 4,822 (56.3) |
| Mobile | 3,538 (41.3) |
| Tablet | 208 (2.4) |

**Legend:** This table lists the number users of *MyGuide Long COVID* by device from August 10, 2023, to August 9, 2024. The percentages represent the proportion of total new users during this period (8,570).

# **Table S4: New users by medium**

| **Medium** | **Definition** | **New Users (%)** |
| --- | --- | --- |
| Direct | User has typed the URL directly into their web browser | 4,383 (51.1) |
| Referral | User has clicked on an existing link on another website | 3,576 (41.7) |
| Organic | User has found MyGuide through a web search engine *or* clicked the link on social media | 550 (6.4) |

**Legend:** This table lists the number users of *MyGuide Long COVID* by medium (i.e., how users found the website) from August 10, 2023, to August 9, 2024. The percentages represent the proportion of total new users during this period (8,559).

# **Table S5: *MyGuide Long COVID* sessions by topic**

| **Topic** | **Sessions** |
| --- | --- |
| Post-exertional Malaise | 1,339 |
| What is Long COVID? | 1,257 |
| Navigating the Healthcare System | 1,081 |
| Pacing | 1.036 |
| Fatigue | 822 |
| Tools and Worksheets | 777 |
| Dysautonomia | 765 |
| Returning to Physical Activity | 670 |
| Brain Fog and Cognitive Issues | 602 |
| Heart Rate Monitoring | 568 |

**Legend:** This table provides the total number of sessions for each topic (a series of pages) between the launch of *MyGuide Long COVID* on August 10, 2023, to August 9, 2024. On *MyGuide Long COVID*, a “topic” is a collection of web pages on a particular long COVID symptom or self-management strategy. A session is defined as a period of time during which a user interacts with the website.
